# *In Situ* Mineralization of Gold Nanoparticles from Sodium Aurothiomalate or Tetrachloroauric Acid in Human Cells

**DOI:** 10.1021/acsnanoscienceau.5c00199

**Published:** 2026-03-10

**Authors:** Muriel F. Gusta, Sofia Rubio, Macarena Cobaleda-Siles, Silvia Pujals, Maria de la Mata, Jordi Arbiol, Neus G. Bastus, Victor Puntes

**Affiliations:** 1 Catalan Institute of Nanoscience and Nanotechnology (ICN2), CSIC and BIST, Campus UAB, Bellaterra, Barcelona 08193, Catalonia, Spain; 2 Vall d’Hebron Institut de Recerca (VHIR), Barcelona 08035, Spain; 3 Networking Research Centre for Bioengineering, Biomaterials, and Nanomedicine (CIBER-BBN), Madrid 28040, Spain; 4 Department of Biological Chemistry, Institute for Advanced Chemistry of Catalonia (IQAC−CSIC), Barcelona 08034, Spain; 5 Dpto. Ciencia de los Materiales, I. M. y Q. I., IMEYMAT, Universidad de Cádiz, Campus Río San Pedro, Puerto Real 11510, Spain; 6 ICREA, Pg. Lluís Companys 23, Barcelona 08010, Catalonia, Spain

**Keywords:** cytoplasmic Au reduction, tetrachloroauric acid, sodium aurothiomalate, cellular oxidative stress, nucleation and growth kinetics

## Abstract

Biological systems can convert ionic gold precursors into nanoparticles (NPs) via redox-driven mineralization, yet the pathways and outcomes remain poorly understood. To address this, we investigated gold nanoparticle (Au NP) spontaneous formation from tetrachloroauric acid (HAuCl_4_) and sodium aurothiomalate (NaAuS) in mammalian cells and a complete cell culture medium. HAuCl_4_ underwent rapid multielectron reduction, producing abundant small intracellular NPs that, at higher concentrations, coalesced into compact clusters and compromised cell viability. In contrast, NaAuS followed slower one-electron reduction, was tolerated at higher concentrations, and yielded fewer but larger assemblies, including fibrous superstructures composed of crystalline Au domains. Extracellular mineralization generated morphologies distinct from those observed intracellularly, indicating that intracellular NPs result from cytoplasmic reduction rather than the uptake of extracellular products. Perturbations of the redox balance suppressed intracellular nucleation and promoted Au reduction in the extracellular milieu. Analysis of the NP size distributions provided mechanistic fingerprints: HAuCl_4_ produced narrow Gaussian-like profiles under synchronized nucleation, whereas NaAuS consistently generated broader log-normal distributions reflecting asynchronous growth. These findings demonstrate that the precursor and cellular redox state can be adjusted to control NP localization and morphology. Overall, this work links precursor chemistry and redox biology to NP architecture and highlights opportunities for biologically guided nanoparticle synthesis in imaging and therapeutic applications.

## Graphical abstract

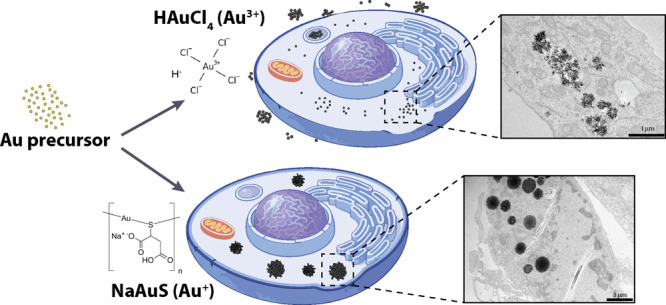

## Introduction

The mineralization of ionic species into inorganic nanoparticles (NPs) is a well-established phenomenon in biological systems. In these biosynthetic processes, biomolecules, including carbohydrates, enzymes, vitamins, and proteins, fulfill a dual role: they can accept and donate electrons to reduce or oxidize metal cations into insoluble species while simultaneously being adsorbed onto the nascent particle surfaces, thereby stabilizing them postnucleation and preventing subsequent aggregation or further growth. Organisms have long exploited this strategy, for example in the biosynthesis of magnetosomes for navigation,^1^ the construction of endo- and exoskeletons,^2^ and the environmental formation of silver NPs driven by humic acids,^3–5^ a phenomenon likewise observed in plants and diverse microorganisms^6^ as a means of detoxification, converting reactive ionic species into inert mineral forms.^7^

While microbial biomineralization has been extensively investigated, research on NP formation within mammalian cells is rapidly expanding.^8^ Within this context, sodium aurothiomalate (NaAuS) represents a particularly relevant and illustrative case. This FDA- and EMA-approved Au­(I) drug has been used for nearly a century in the treatment of rheumatoid arthritis,^9–16^ yet its mechanism of action remains partially understood. Clinical observations, including dermal chrysiasis and the appearance of purple-colored urine,^17–19^ suggest the *in situ* conversion of ionic Au precursors into Au NPs. Au NPs are rapidly coated by a protein corona in physiological conditions^20,21^ and tend to either accumulate in the liver prior to hepatobiliary excretion or to be eliminated through the urinary system depending on their size.^17–19^ These findings indicate that the pharmacological activity of Au­(I) compounds may depend not only on the ionic species activity but also on their intracellular transformation into Au NPs under physiological conditions. This conceptual framework reconciles clinical observations^8^ and highlights the potential for the rational redesign of gold-based therapeutics to direct NP formation toward specific tissues or pathological niches.^22^

Recent studies have demonstrated the intracellular formation of gold nanoparticles (Au NPs) from ionic gold species in various mammalian cell types, as well as in lysed cells providing a reducing environment for noble metal cations.^7,23–25^ It has been shown that internalized gold, introduced either as ionic species or as preformed NPs, can converge toward electron-dense lysosomal gold deposits historically termed aurosomes, originally described more than five decades ago in the context of chrysotherapy.^26,58–62^ Aurosomes appear to represent a common intracellular end point for gold biotransformation, largely independent of the initial chemical form of administration. This convergence bridges classical observations from chrysotherapy with modern gold nanomedicine and suggests that gold-based therapies should be viewed within a unified biological framework governed by intracellular redox chemistry and compartmentalization.

Mechanistically, proteins present in the extracellular milieu, either derived from complete cell culture media (cCCM) or secreted by the cells themselves, can promote the initial partial reduction of soluble Au­(III) or Au­(I) ions.^27^ The remaining ionic species are then able to cross the plasma membrane, likely via copper transporters, as observed for other metal cations,^28,29^ or through complexation with proteins.^30,31^ Once within the cytoplasm, the stronger reducing environment provided by biomolecules, such as glutathione, thioredoxin, NADPH, and other redox-active species, drives their conversion into metallic gold.^25^

This biosynthetic pathway enables direct Au NP formation within the cytosol, an essential feature for their exploitation in imaging and therapeutic applications, effectively bypassing endocytosis and vesicular entrapment,^32–35^ an essential feature for their exploitation in surface-enhanced Raman spectroscopy (SERS),^36,37^ fluorescence,^7,38^ computed tomography,^39^ and photothermal ablation.^40^ Intracellular Au NP formation can also be exploited to enhance radiotherapy.^41^ Upon irradiation, owing to their high atomic number, these particles efficiently absorb high-energy photons, releasing secondary electrons and catalyzing the generation of reactive oxygen species (ROS), thereby amplifying the therapeutic effect.^42^ In addition, these NPs are generated *in situ* without additional surfactants and, therefore, display superior biocompatibility compared with chemically synthesized counterparts.^43–45^ Although both intra- and extracellular formation of Au NPs has been documented, their successful biomedical translation requires precise control over nucleation and growth.

Several important questions remain unresolved. One is whether different precursors give rise to distinct intracellular morphologies, such as well-dispersed NPs or aggregated superstructures, and how these morphologies relate to their underlying formation mechanisms. A second question is whether the NPs observed within cells truly originate from cytoplasmic mineralization or instead result from the uptake of particles preformed in the extracellular media. Finally, the statistical characteristics of NP size distributions may serve as mechanistic fingerprints of nucleation and growth pathways, indicative of cell status.

To this end, we investigated the intra- and extracellular biosynthesis of Au NPs from two distinct precursors under varying conditions: (i) tetrachloroauric acid (HAuCl_4_), a classical reagent for colloidal Au NP synthesis,^46,47^ and the most commonly reported precursor in studies of intracellular Au NP formation, known for its high reactivity and cytotoxicity. This allows for a direct comparison with the existing literature, and (ii) sodium aurothiomalate (NaAuS), a clinically approved Au­(I)-based drug with a long history of therapeutic use in rheumatoid arthritis. We exposed both precursors to cCCM and different mammalian cell types to assess the influence of cellular reductive biology, under both homeostatic and oxidative stress states, on NP formation, localization, and morphology.

## Results

To investigate the Au mineralization process, HeLa human cervical cancer epithelial cells were selected as a model system owing to their robust growth, well-characterized redox status, and reproducible responses to oxidative stress,^48,49^ a hallmark of inflammation. Cells were incubated with either HAuCl_4_ or NaAuS at concentrations ranging from 100 to 1000 μM for 24 h. Lower concentrations (∼167 μM) correspond to typical NP synthesis conditions, whereas higher concentrations (up to 2.5 mM) approximate therapeutic drug levels of Au­(I)-based antirheumatic drugs, such as Auranofin.^50,51^ A 24 h exposure period was chosen to ensure sufficient cellular uptake. Following incubation, cells were fixed with glutaraldehyde, embedded in epoxy resin, and sectioned into 60 nm ultrathin slices for ultrastructural characterization by transmission electron microscopy (TEM).

Obtained results indicate that HAuCl_4_ and NaAuS produce distinct outcomes (Figure 1A,B). HAuCl_4_ generated numerous small NPs (∼4 nm), consistently observed both attached to the external cytoplasmic membrane and freely dispersed throughout the cytoplasm. At the lower concentrations (100–250 μM), these NPs form large, compact intracellular aggregates, a morphology characteristic of rapid burst nucleation driven by the high reactivity of Au­(III). At the highest concentration tested (1000 μM), cellular uptake was impaired due to cell toxicity, and deposits accumulated primarily in the extracellular space. At intermediate concentrations (250–500 μM), Au deposits could be found at the cytoplasmic membrane, inside the cell within vesicles, and freely dispersed throughout the cytoplasm (Figure S1). By contrast, when employing NaAuS as a precursor, fewer NPs, substantially larger than those derived from HAuCl_4_, were found (Figure 1B and Figure S2). In this case, at concentrations of 250 μM and above, elongated linear chains of NPs appeared, which later fused into sponge-like aggregates made of small (ca. 3 nm) Au NPs.

**Figure 1:**
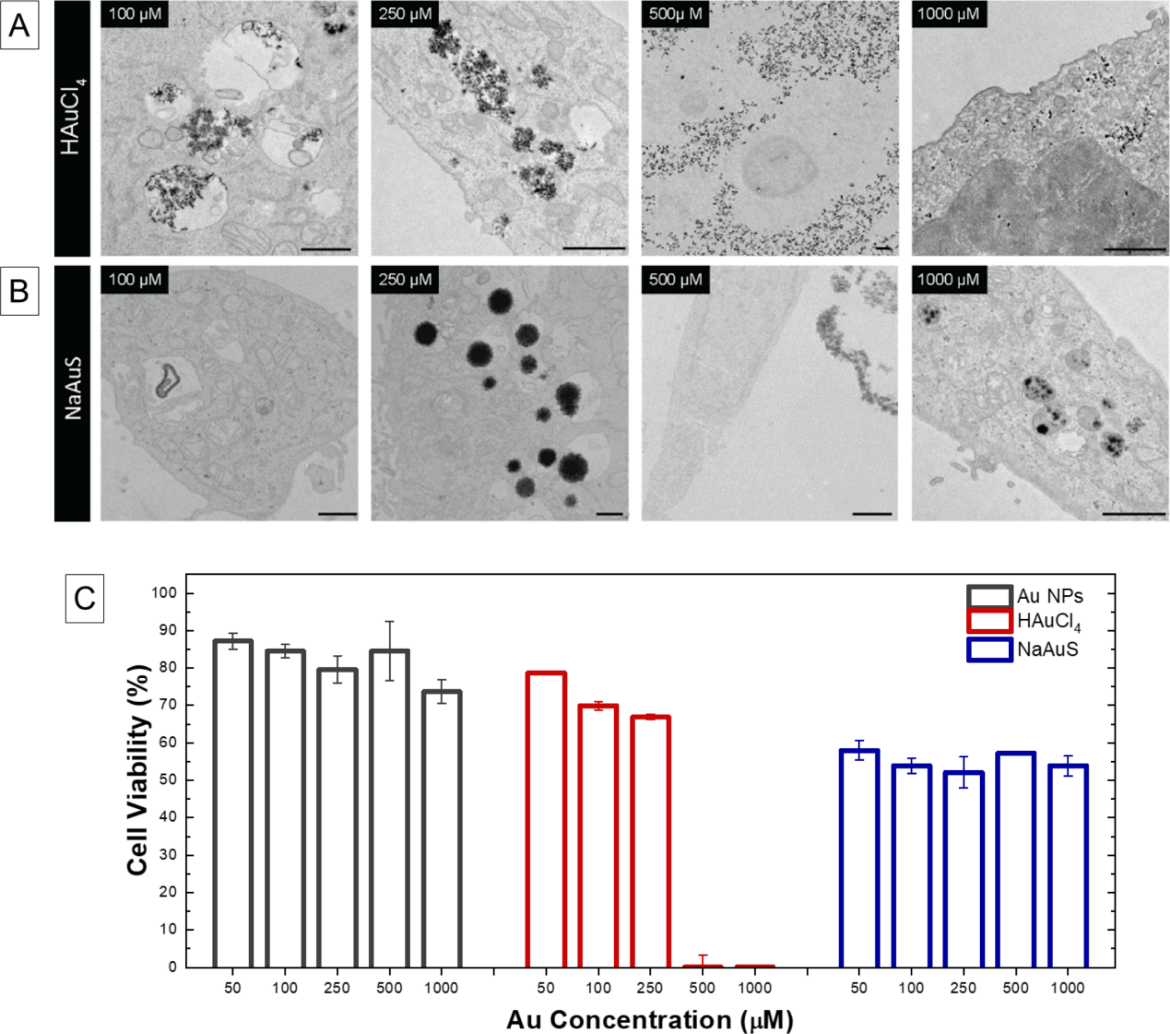
Representative bright-field transmission electron microscopy (TEM) images of the intracellular formation of Au NPs resulting from the exposure of (A) HAuCl_4_ and (B) NaAuS to HeLa cells at various precursor concentrations (100, 250, 500, and 1000 μM) during a 24 h incubation period. (C) PrestoBlue cell viability assay of the HAuCl_4_ and NaAuS precursors and gold nanoparticles at the same Au relative concentrations (50–1000 μM).

Cell viability assays complemented these observations, revealing a steep concentration-dependent decrease in survival for HAuCl_4_-treated cells (Figure 1C). Because each Au^3+^ ion requires three electrons for its reduction, this process consumes substantial amounts of intracellular reductants, such as glutathione and thioredoxin, disrupting redox homeostasis and ultimately compromising cell viability. In the case of NaAuS, even at the highest concentration tested (1000 μM), approximately 60% of cells remained viable, reflecting the greater chemical stability of NaAuS and the lower redox stress it induces. As an additional control, citrate-stabilized presynthesized Au NPs (Figure S3) were incubated with cells at equivalent concentrations. In all cases, the preformed NPs exhibited negligible toxicity, confirming both, the absence of Au NPs free in the cytoplasm and that the cytotoxic effects observed with HAuCl_4_ and NaAuS arise from the chemical reactivity of the ionic precursors and their intracellular reduction,^52^ rather than from the NPs themselves once formed.^53–55^

Au NPs synthesized within live cells were recovered following cell lysis (Figure S4). Regardless of the lysis method used, lysates from HAuCl_4_-exposed cells consistently displayed uniform ∼4 nm NPs (Figure S4A,B), in agreement with earlier reports.^56^ In contrast, lysates obtained from NaAuS-exposed cells (Figure S4C) yielded more polydisperse populations prone to aggregation, with individual particles even larger than those observed in intact cells (Figure 1B). Because these morphologies were absent in intact cells, they are interpreted as artifacts of the lysis process during extraction and purification, likely reflecting the continued reduction of residual Au­(I), which further grows and reorganizes into larger NPs. Interestingly, these features resemble protoparticle intermediates reported in citrate-mediated Au NP synthesis, which are enriched in Au­(I) and stabilized by organic ligands.^46,57^

A particularly remarkable outcome of NaAuS exposure was the intracellular formation of fibrous Au superstructures. At a 250 μM concentration, elongated linear NP chains fused into sponge-like aggregates (Figure 1B). TEM overview images revealed large, spherical aggregates with rugged, spiky peripheries embedded within the cytoplasm (Figure 2A–F). At higher magnification, these structures resolved into networks of NP chains rather than compact NPs. High-resolution TEM confirmed that these structures were composed of ∼2.7 nm crystalline Au domains, with lattice fringes of 2.35 Å corresponding to the (111) planes of face-centered cubic gold (Figure 2G,H). Fast Fourier transform (FFT) analysis further confirmed the crystalline order (Figure 2I). Angular dark-field scanning transmission electron microscopy (ADF-STEM) provided a clear Z-contrast, distinguishing the dense gold phase from the lighter organic matrix (Figure 2J). In this mode, Au crystals appeared as bright spots embedded in a low-contrast intracellular matrix. Higher-magnification ADF-STEM images corroborated the rugged fibrous morphologies observed by TEM (Figure 2K,L). STEM energy-dispersive X-ray spectroscopy (EDS) elemental mapping of selected samples (Figure 2M,O) verified that the bright fibrous assemblies were predominantly composed of Au embedded in a carbon-rich matrix (Figure 2N,P), with large-area EDS spectra further confirming this composition (Figure S5). Similar processes have been reported in chrysiasis, where macrophages have been observed to contain both amorphous *aurosomes* and crystalline deposits.^26,58–62^ Furthermore, NaAuS has been shown to exhibit strong affinity for cytoskeletal and cytoskeleton-associated proteins, implying that these interactions may provide structural templates guiding the formation of fibrous Au superstructures.^63^ By contrast, the rapid reduction of Au­(III) bypasses intermediate stabilization, resulting in the direct formation of compact clusters.

**Figure 2:**
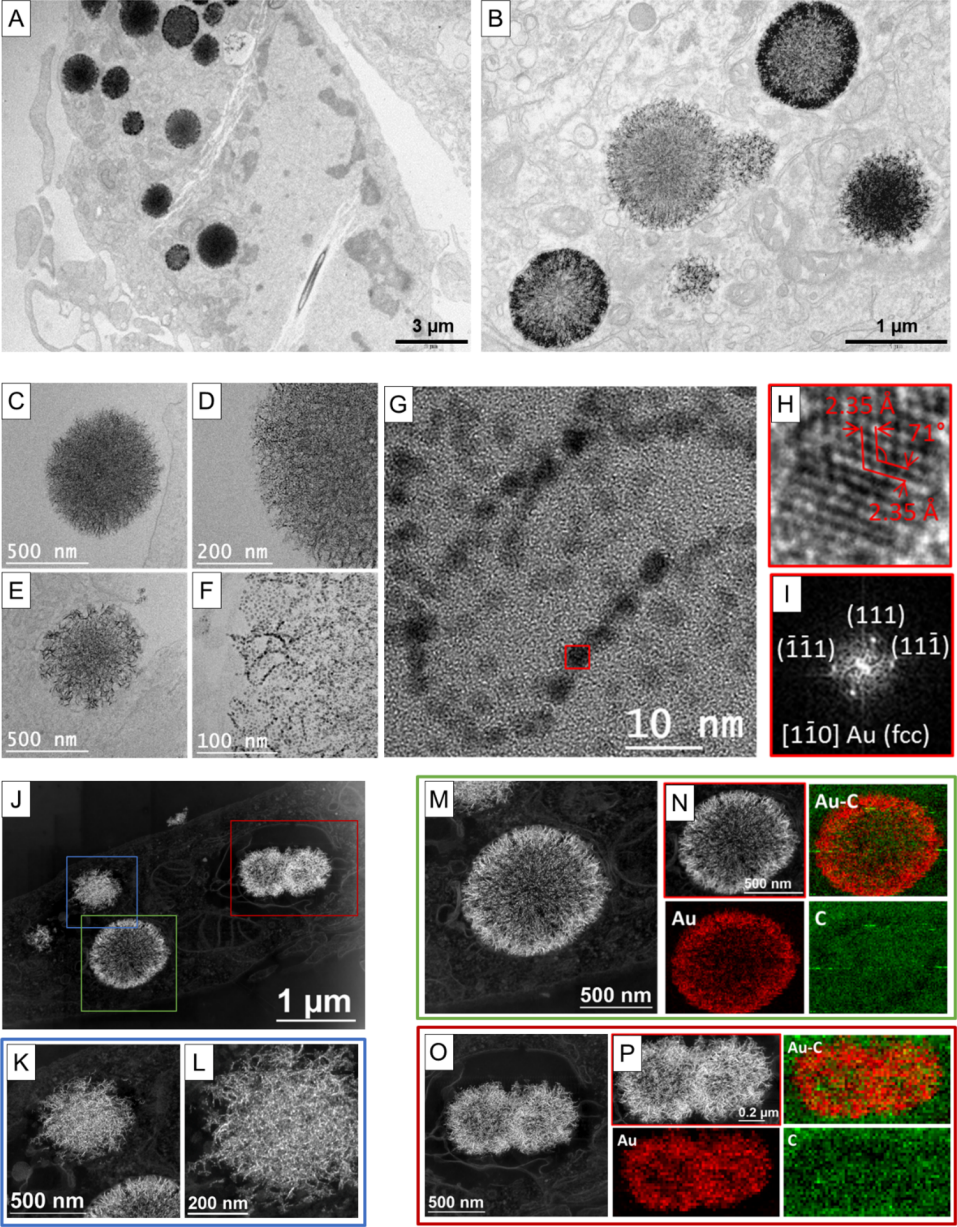
(A, B) Representative bright-field (BF) TEM images of the Au nanostructures formed by the reduction of 250 μM NaAuS. (C–F) Higher-magnification TEM images show greater detail of these nanostructures that consist of strands of spheroidal nanoparticles. (G) HR-TEM detail showing the crystallinity of the spheroidal nanoparticles with a mean diameter of 2.7 nm. (H) Magnified detail of the red square nanoparticle in panel (G), with the corresponding crystal structure analysis (I), revealing that the small nanoparticle could be unambiguously assigned to cubic fcc elemental Au (metallic). The atomic plane distance between (111) of Au is 2.35Å, and the angle between them is 71°. (J) ADF-STEM micrograph of the Au nanostructures in the cell formed by the reduction of NaAuS. Square regions are magnified in panels (K) and (L) (blue), (M) (green), and (O) (red). (N, P) EDX maps of the selected regions: elemental Au (in red) and C (in green) maps and color composite map for Au–C.

To discern Au NPs synthesized intracellularly from those formed extracellularly and subsequently internalized, HAuCl_4_ and NaAuS were incubated in cCCM, consisting of 90% Dulbecco’s modified Eagle medium (DMEM) supplemented with 10% fetal bovine serum (FBS), at a final concentration of 500 μM and 37 °C. Aliquots were collected at 24, 48, and 64 h, as well as at 7 and 15 days, and analyzed by TEM and UV–vis spectroscopy. These results show that in addition to intracellular mineralization, Au precursors can undergo reduction in the extracellular medium, a distinction that is critical because cells are exposed not only to ionic precursors but also to any NPs already formed in the culture media.

Representative TEM images (Figure 3) demonstrate the systematic formation of Au NPs (see Table S1 for the detailed composition). The final NP morphology was strongly dependent on the employed precursor, reflecting the precursor-specific behaviors observed in cells. HAuCl_4_ produced small, spherical NPs within 24 h (Figure 3A). These NPs coalesced into larger, irregular, polycrystalline flower-like aggregates with longer incubation times (48–64 h), which appear further densified after 15 days through particle coalescence and monomer incorporation. By contrast, after 24 h, NaAuS yielded only diffuse, low-contrast aggregates, lacking the well-defined spherical NPs produced by HAuCl_4_ (Figure 3B). By 48–64 h, these assemblies began to display discrete crystalline spots within an amorphous matrix, consistent with delayed nucleation of small Au nanocrystals. Over time, irregular porous networks formed, and by day 15, the dominant structures were heterogeneous sponge-like aggregates. Dark-field TEM confirmed the polycrystalline nature of both types of aggregates (Figure S6). Compared with the compact, flower-like assemblies formed from HAuCl_4_, aggregates formed in the presence of NaAuS remained less dense and poorly organized, reflecting the slower reduction kinetics of Au­(I) precursors. Crystallinity was further confirmed by selected area electron diffraction (SAED), which showed patterns consistent with face-centered cubic (fcc) Au, and by electron probe microanalysis (EPMA), which verified their Au elemental composition (Figure S7).

**Figure 3:**
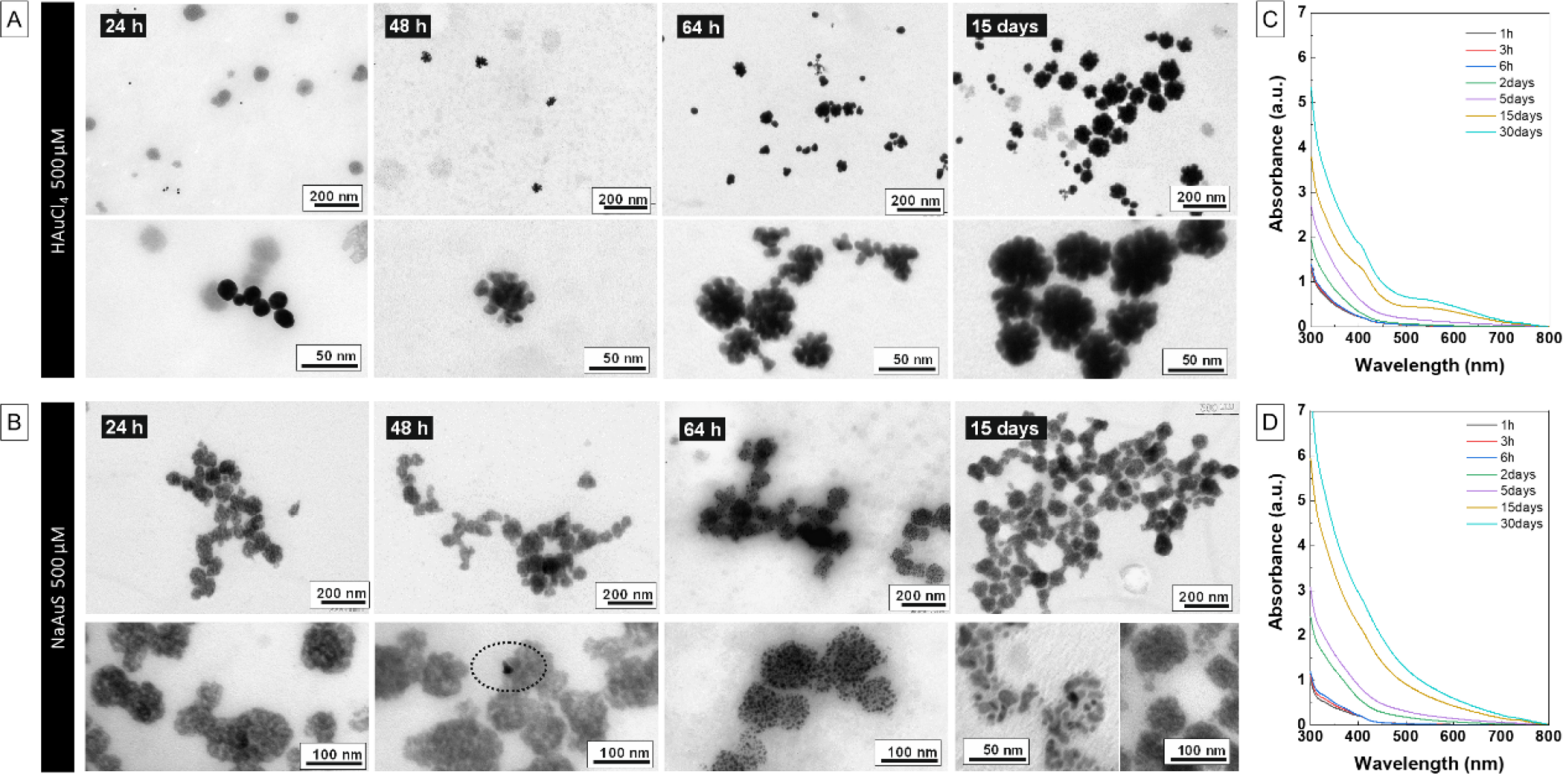
Representative bright-field transmission electron microscopy (TEM) images of Au NPs following exposure of HAuCl_4_ (A) and NaAuS (B) to cCCM at a final concentration of 500 μM. The incubation was conducted at 37 °C for 24 h, 48 h, 64 h, and 7 days. The temporal evolution of UV–vis absorption spectra for HAuCl_4_ (C) and NaAuS (D) in cCCM at a final concentration of 500 μM.

The temporal evolution of the Au precursor reduction in cCCM was also monitored by UV–vis spectroscopy. These extracellular matrix experiments are critical for distinguishing the origin of the NPs. Across all conditions, absorbance increased over time, reflecting both progressive Au NP formation and cCCM aging, as indicated by the gradual rise in background absorbance in the whole UV–vis region. For HAuCl_4_, the growth and aggregation of particles manifested as a broad surface plasmon resonance (SPR) band in the 500–600 nm range^64^ (Figure 3C). Especially at elevated HAuCl_4_ concentrations (1000 μM–2.5 mM), the SPR band became more intense and well-defined, enabling precise monitoring of NP formation even at shorter incubation times. In contrast, NaAuS did not display a distinct SPR peak, likely due to its slower reduction kinetics, which produced fewer and more aggregated Au NPs, resulting in plasmon damping (Figure 3D). Importantly, no NP formation was observed when either precursor was incubated in Milli-Q water, confirming that Au reduction was mediated by biomolecules present in cCCM (Figure S8).

Concentration-dependent studies (100 μM to 2.5 mM) revealed consistent trends across the full range of precursor concentrations (Figure S9). While both HAuCl_4_ and NaAuS can be reduced by biomolecules in cCCM to form Au NPs, the morphologies generated extracellularly—dense flower-like aggregates for HAuCl_4_ and fluffy, heterogeneous assemblies for NaAuS (Figure 3)—differ markedly from those observed inside intact cells (∼4 nm spherical clusters, sponge-like aggregates, or fibrous superstructures; Figures 1 and 2). This contrast demonstrates that the intracellular NPs imaged by TEM are not merely internalized extracellular products but result from mineralization processes occurring within the cellular environment. Consequently, precursor chemistry and subcellular location together dictate the pathway of Au reduction, leading to distinct extracellular versus intracellular structures.

Next, we examined how the reduction of Au cations and the resulting NPs depend not only on precursor chemistry and concentration but also on the cellular redox state, which is determined by intracellular ROS levels and the availability of antioxidant enzymes, such as superoxide dismutase (SOD) and thioredoxin, and small molecules, such as glutathione and ascorbic acid.^65,66^ To investigate this, HeLa cells were pretreated with *tert*-butyl hydroperoxide (TBH), a well-established inducer of oxidative stress that elevates intracellular ROS levels and mimics pro-inflammatory conditions,^67,68^ and then exposed to HAuCl_4_, whose three-electron reduction imposes a strong demand on intracellular reducers. Following TBH treatment, cells were incubated with 100 μM HAuCl_4_ and analyzed by TEM and confocal laser scanning microscopy (CLSM). CLSM in reflectance mode proved particularly valuable for detecting NP aggregates larger than 50 nm with minimal sample processing.^69^ In untreated HeLa cells, TEM revealed abundant NP aggregates that dispersed throughout the cytoplasm (Figure 4A). Additionally, CLSM confirmed their intracellular location, showing red reflectance signals confined within the green CellMask-stained membranes (Figure 4D).

**Figure 4:**
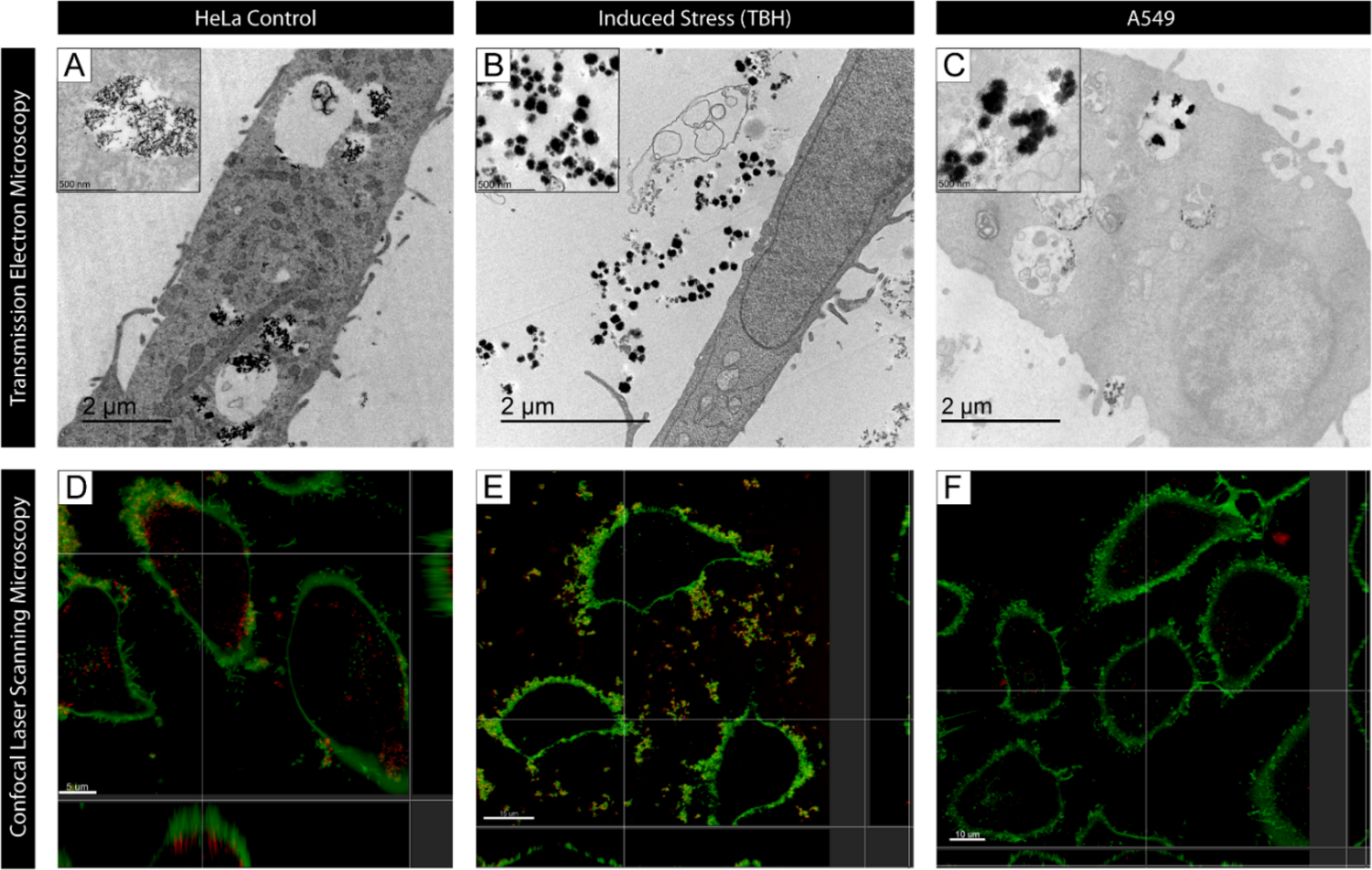
Effect of the redox cell state on cytoplasmic reduction of gold was studied by comparing the formation of NP in cells with different ROS basal levels: high for TBH-exposed HeLa cells and low for the A549 cell line. (A–C) Representative bright-field TEM images after a 24 h incubation with 100 μM HAuCl_4_. (D–F) Representative CLSM images. The cell membrane was stained with CellMask (green), and NP was imaged in reflectance mode (red). The orthogonal views from the acquired Z-stack allow us to confirm the intracellular localization of Au nanostructures.

In contrast to untreated cells, TBH-stressed HeLa cells exhibited no intracellular formation of Au NPs, suggesting that TBH depletion of intracellular reductants lowers the reducing capacity below the threshold necessary for Au^3+^ nucleation and oxidizes glutathione and other intracellular antioxidants, depleting from available reducers and thereby preventing intracellular NP nucleation. Instead, NPs accumulated exclusively in the extracellular medium (Figure 4B), consistent with Au^3+^ reduction by serum components. CLSM confirmed this finding, showing the absence of intracellular signals and the presence of red reflections associated with extracellular Au aggregates (Figure 4E). A similar trend was observed in A549 cells, which have lower basal ROS levels and reduced antioxidant enzyme activity compared with HeLa cells (Figure 4C,F).^70,71^ TEM revealed only sparse deposits, and CLSM confirmed the near absence of cytoplasmic NP signals. The limited nucleation is explained by the low concentration of antioxidants, most notably glutathione, which diminishes the ability of these cells to reduce Au^3+^ and alters the morphology of the Au NP structures formed.^71,72^ These observations demonstrate that both excessive oxidative stress (TBH-treated HeLa cells) and intrinsically low antioxidant capacity (A549 cells) prevent efficient intracellular mineralization. This mechanistic link suggests that the biological effects of Au compounds are closely tied to their redox-dependent reduction into NPs and that investigating NP synthesis dynamics in different cellular redox environments may provide critical insights into their therapeutic activity. In particular, the anti-inflammatory effects of gold drugs may arise from their conversion into NPs, which in turn modulate ROS levels in inflamed tissues.^73–75^

## Discussion

Nanoparticle formation in biological environments follows the principles of nucleation and growth that govern colloidal chemistry, although the cellular context adds additional complexity. In classical colloidal chemistry, NP formation is described by the LaMer model,^76^ in which a burst of supersaturation-driven nucleation is followed by slower, diffusion-limited growth. Once supersaturation of gold precursors is reached, the kinetics of nucleation determines the particle size and distribution. Rapid, burst nucleation consumes monomers quickly, producing many small, relatively uniform NPs, whereas slower or prolonged nucleation favors growth on pre-existing nuclei, yielding fewer but larger particles.^46,77^ The degree of synchronization between these stages determines the resulting particle size distributions. Rapid, simultaneous nucleation yields symmetric Gaussian-like distributions, whereas prolonged or asynchronous nucleation produces log-normal profiles. Applying this framework to Au mineralization reveals how precursor chemistry, concentration, and cellular redox balance dictate whether NPs remain small and monodisperse or assemble into larger superstructures.

Figure 5 compiles the size frequency distributions of the obtained structures under all experimental conditions, with optimal statistical fittings and *R* ^2^ values provided in Table 1. For HAuCl_4_, highly reactive Au^3+^ ions drive synchronized nucleation at low concentrations, but increasing precursor concentration and associated cytotoxicity progressively disrupt this regime. At 100 μM (Figure 5A), the distribution approximates a Gaussian, with a ∼4 nm population representing the minimum nucleation size and serving as a fingerprint of rapid Au^3+^ reduction. A slightly asymmetric tail toward larger diameters indicates early aggregation and deviation from an ideal Gaussian. As the concentration increased (Figure 5B,C), this asymmetry became more pronounced. At 1000 μM, severe cytotoxicity nearly abolished intracellular reduction, leaving only a narrow fraction of small NPs. The disappearance of larger particles reflects the exhaustion of intracellular reducing capacity, preventing further growth and aggregation.

**Figure 5:**
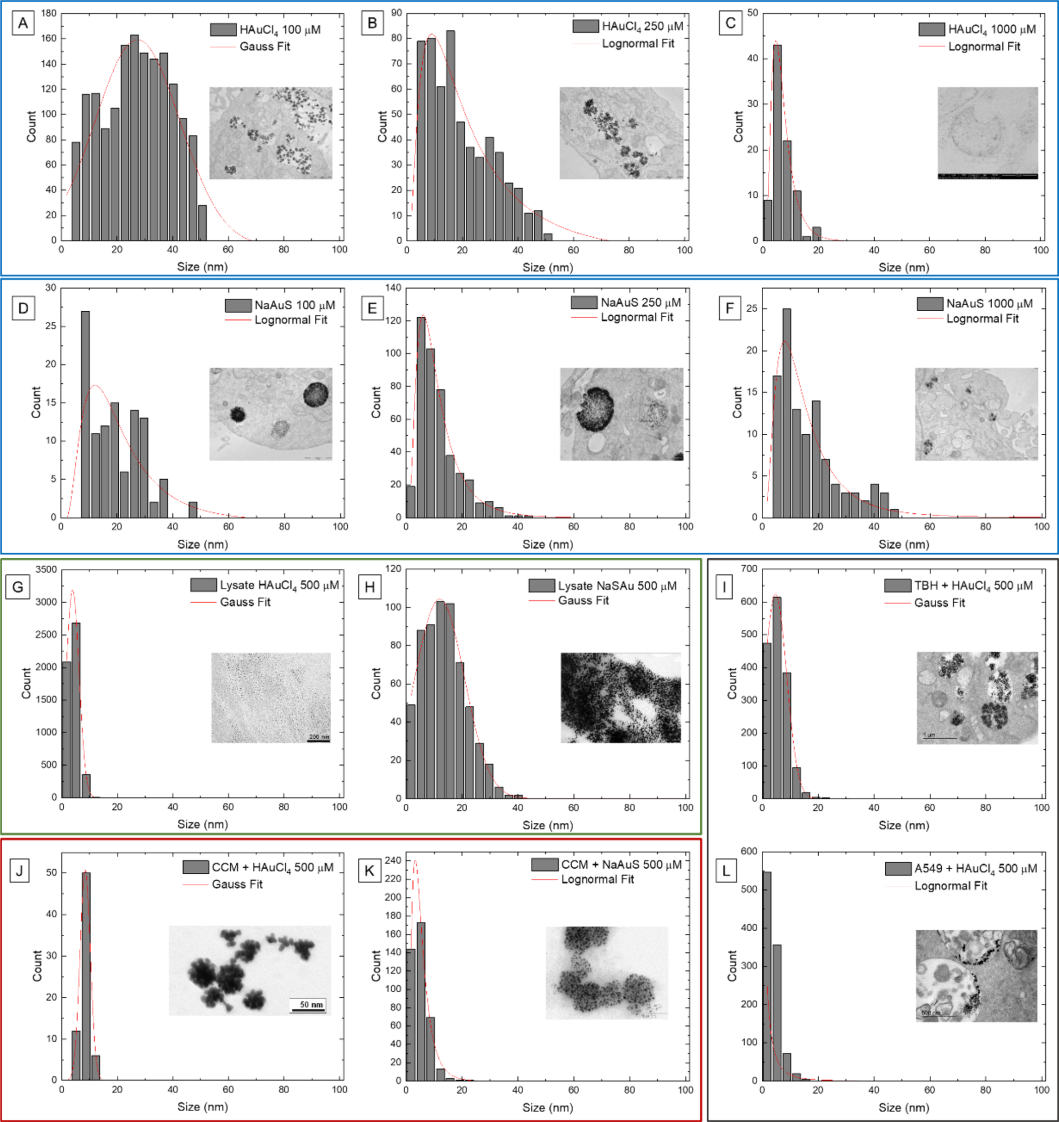
Size frequency distribution analysis from the structures detailed in the insets. (A–C, blue) HeLa cells exposed to 100, 250, and 1000 μM HAuCl_4_. (D–F, blue) HeLa cells exposed to 100, 250, and 1000 μM NaAuS. (G, H, green) NPs recovered through cell lysis after exposure of HeLa cells to 500 μM HAuCl_4_ and NaAuS (J, K; red) Au NPs following exposure of HAuCl_4_ and NaAuS to cCCM at a final concentration of 500 μM. (I, L; gray) Au NPs from TBH-preexposed HeLa cells and A549 cells after exposure to 100 μM HAuCl_4_. Colors are used to group graphs based on key differences in localization of Au reduction, metabolism, or sample postprocessing.

**Table 1:** Mean Size of Observed Au Structures, and Corresponding *R* Values from Statistical Fittings of Size Frequency Distributions Shown in Figure 5 ^a^

reducing environment	Au precursor	Au (μM)	sample analyzed	figure reference	mean size (nm)	*R*-adj Gauss fit	*R*-adj Lorentz fit	*R*-adj log-normal fit
HeLa cells	HAuCl_4_	100	Au in cells at 24 h	Figure 1A, Figure S1	27.1 ± 12.2	0.9112*	0.88157	0.75457
HeLa cells	HAuCl_4_	250	Au in cells at 24 h	Figure 1A, Figure S1	19.5 ± 11.7	0.7734	0.80041	0.92826*
HeLa cells	HAuCl_4_	1000	Au in cells at 24 h	Figure 1A, Figure S1	7.2 ± 3.6	0.95335	0.98455	0.99337*
HeLa cells	NaAuS	100	Au in cells at 24 h	Figure 1B, Figure S2	19.6 ± 9.2	0.58889	0.55338	0.66812*
HeLa cells	NaAuS	250	Au in cells at 24 h	Figure 1B, Figure S2	11.4 ± 7.1	0.8999	0.91693	0.99356*
HeLa cells	NaAuS	1000	Au in cells at 24 h	Figure 1B, Figure S2	16.7 ± 10.7	0.7532	0.79285	0.91535*
HeLa cells	HAuCl_4_	500	Au recovered from cell lysate	Figure 2A	4.0 ± 1.8	1*	0.99843	0.99989
HeLa cells	NaAuS	500	Au recovered from cell lysate	Figure 2C	13.7 ± 7.6	0.99233*	0.97767	0.90324
HeLa cells + TBH	HAuCl_4_	500	Au in cells at 24 h	Figure 5B	5.8 ± 3.3	0.999*	0.97869	0.98047
A549 cells	HAuCl_4_	500	Au in cells at 24 h	Figure 5C	3.8 ± 2.5	–0.11	0.43059	0.49205*
cCCM	HAuCl_4_	500	Au in cCCM	Figure 3A	8.3 ± 1.5	1*	0.9956	0.99997
cCCM	NaAuS	500	Au in cCCM	Figure 3B	5.1 ± 2.8	0.99957	0.9918	0.99681*

a In bold and marked with (*) are highlighted the best *R* values fitted in the size distribution graphs in Figure 5.

In contrast, NaAuS consistently produced log-normal distributions across all concentrations (Figure 5D–F), prolonging the nucleation window and generating heterogeneous populations, consistent with diffusion-driven growth. Unlike HAuCl_4_, NaAuS maintained broad, asymmetric distributions even at high concentrations, as its slower kinetics and lower redox demand supported continued growth. At 100 μM, the distribution was broad with small nuclei coexisting alongside larger aggregates. At 250 μM and above, the distributions broadened further, and TEM confirmed the presence of sponge-like and chain-like assemblies, consistent with ongoing nucleation and aggregation.

The biological environment further reinforced this behavior. High ionic strength screened electrostatic repulsion, promoting aggregation, while abundant proteins stabilized individual particles or bridged them into clusters.^20,21,64,78^ These interactions amplified subtle differences in particle size or surface chemistry, broadening distributions and shifting them toward larger diameters and affecting the ultimate intracellular localization of Au NPs. The precursor-dependent signatures were also observed in particles recovered from cell lysates and the extracellular medium. In these conditions, NPs derived from HAuCl_4_ consistently exhibited narrow, ∼4 nm Gaussian-like distributions (Figure 5G,H), whereas NaAuS lysates yielded larger, more polydisperse log-normal populations, reflecting slower and heterogeneous growth. Analysis of NP size distributions provided a mechanistic fingerprint of the formation processes. HAuCl_4_ favored narrow, Gaussian-like distributions under synchronized reduction, while NaAuS consistently produced broader log-normal distributions, reflecting asynchronous nucleation and aggregation. These statistical signatures were preserved in both cell lysates and extracellular media formed products (Figure 5J,K), indicating that distribution shape captures nucleation history and serves as a sensitive readout of the redox environment and cellular health status. HAuCl_4_ produced compact Gaussian-like populations, whereas NaAuS generated broad, log-normal ensembles centered on small nanocrystals embedded in fluffy, low-density assemblies, consistent with a prolonged, heterogeneous growth pathway. These factors collectively explain the observed differences between extracellular and intracellular NP distributions.

Perturbations of the cellular redox balance further reinforced this principle. In TBH-pretreated HeLa cells (Figure 5I), oxidative stress prevented intracellular nucleation, shifting Au reduction to the extracellular milieu, yielding broad and heterogeneous distributions that closely resembled those observed for NaAuS. Similarly, in A549 cells (Figure 5L), which are characterized by intrinsically lower antioxidant capacity, NaAuS nucleation was inefficient and delayed, again producing broad, polydisperse populations. These findings demonstrate that both extremes of redox imbalance—excessive ROS or insufficient reductive power—disrupt synchronized nucleation and broaden NP populations. Importantly, the preservation of these statistical signatures in cell lysates and extracellular medium confirms that the distribution shape reflects the nucleation history rather than being altered by extraction. The results demonstrate that NP size distributions serve as a mechanistic fingerprint of precursor reactivity, cellular redox state, and nucleation dynamics. Gaussian-like profiles indicate conditions of efficient, synchronized reduction, whereas log-normal profiles reflect asynchronous nucleation, aggregation, and redox imbalance. Beyond characterizing NP populations, these size distribution statistics provide a powerful framework for deciphering the interplay between precursor chemistry and cellular physiology.

## Conclusions

In this work, gold precursors were exposed to a complete culture medium (cCCM) and mammalian cells to investigate their conversion into gold nanoparticles (Au NPs) through the intrinsic reductive biology of living systems. Both HAuCl_4_ and NaAuS underwent mineralization, but the morphology, localization, and biological impact of the resulting NPs depended strongly on precursor chemistry, concentration, and cellular redox balance. HAuCl_4_, a highly reactive Au­(III) salt, promoted rapid, synchronized nucleation that not only generated abundant ∼4 nm intracellular NPs but also imposed substantial cellular stress and toxicity. In contrast, NaAuS, an Au­(I) compound with slower one-electron reduction kinetics, produced fewer but larger assemblies, including chain-like aggregates, sponge-like morphologies, and fibrous superstructures, while being tolerated at higher concentrations. The fate of biomineralized gold was governed by three interconnected processes: diffusion of ions through the cellular environment, their reduction to metallic Au, and the stabilization and transport of the resulting NPs. Gold ions readily penetrated tissues but were initially cytotoxic due to their strong oxidative potential. Over time, however, they were reduced to bioinert metallic NPs that were more biocompatible and retained potential diagnostic and therapeutic functionalities. Notably, intracellular ROS levels and redox enzyme activity played a decisive role in Au reduction. Balanced redox conditions favored synchronized intracellular nucleation, whereas oxidative stress or intrinsically low antioxidant capacity suppressed mineralization and shifted Au reduction toward the extracellular milieu. Extracellular mineralization in cCCM also produced Au NPs, but with morphologies distinct from those formed within cells, confirming that intracellular NPs observed by TEM are genuine products of cytoplasmic synthesis rather than uptake artifacts. Because of the interplay between precursor reactivity, redox biology, and the physicochemical environment, the statistical characteristics of NP size distributions provide a mechanistic fingerprint of cytoplasmic synthesis pathways. Overall, these findings demonstrate that precursor chemistry, dosage, and redox balance can be manipulated to control whether small, well-dispersed intracellular NPs or larger extracellular aggregates are produced. Beyond providing fundamental insight, this work suggests that biological metabolism itself may be harnessed as a programmable nanofoundry, where precursor design and redox modulation direct the *in situ* architecture of NPs, offering promising opportunities for therapeutic applications, such as imaging, radiosensitization, and hyperthermia.

## Experimental Section

### Chemicals

Gold­(III) chloride trihydrate (HAuCl_4_·3H_2_O), sodium aurothiomalate hydrate (C_4_H_4_AuNaO_4_S, NaAuS), heat-inactivated fetal bovine serum (FBS), glutaraldehyde, sodium phosphate dibasic (Na_2_HPO_4_), and sodium phosphate monobasic (NaH_2_PO_4_) were purchased from Sigma-Aldrich. Dulbecco’s modified Eagle medium (DMEM), fetal bovine serum heat-inactivated (FBS), penicillin–streptomycin (Pen-Strep), and CellMask plasma membrane stain were purchased from ThermoFisher. All chemicals were used as received without further purification. Distilled water passed through a Millipore system (ρ = 18.2 MΩ) was used in all experiments.

### Extracellular Synthesis

HAuCl_4_ (from 100 to 1000 μM) and NaAuS (500 μM) were incubated in complete cell culture media (cCCM), consisting of DMEM (see Table S1 for full composition) supplemented with 10% FBS and kept in an incubator at 37 °C. Aliquots were extracted after 24, 48, and 64 h for their characterization. For TEM analysis, 10 μL aliquots were directly drop-cast onto copper TEM grids and air-dried without prior filtration or centrifugation to preserve the full population of gold-containing species, including highly aggregated structures. UV–vis spectra were recorded on a Shimadzu UV-2400 spectrophotometer using 1 mL samples in a cuvette, collected from 300 to 800 nm at RT, with Milli-Q water as the reference baseline. No additional baseline subtraction was applied.

### Electron Microscopy (EM)

For TEM imaging, samples were visualized using 80 keV TEM (Jeol 1010, Japan) and 20 keV STEM (FEI Magellan 400L XHR SEM). For HR-TEM, ADF-TEM and EDX samples were visualized using 200 kV field emission gun (FEG) high-resolution and analytical TEM/STEM (FEI Tecnai G^2^ F20).

### Cell Culture

HeLa and A549 cell lines were maintained in culture in 75 cm^2^ tissue culture flask using complete cell culture media (cCCM), consisting of DMEM (see Table S1 for full composition) supplemented with 10% FBS and 1% Pen-Strep at 37 °C and humidified 5% CO_2_.

### Intracellular Synthesis

Cells were seeded to 1 × 10^5^ cell/mL on a 10 mm Petri dish during 24 h before exposure. Then, HAuCl_4_ and NaAuS at desired concentrations were added to the cell culture and incubated for 24 h. Sample pellet was fixed in 2% paraformaldehyde, 2.5% glutaraldehyde in 0.1 M phosphate buffer, and incubated at 4 °C for 30 min in a shaker. After centrifugation at 2500 rpm for 5 min, the samples were washed at 4 °C 10 min in the fixation buffer and washed four times for 10 min with PB 0.1 M, pH 7.4 at 4 °C. Then, pellets were washed 4 times for 10 min with double-distilled water at 4 °C. After dehydrating the sample with increasing concentrations of acetone, infiltration into the Spurr resin was performed followed by polymerization. Ultrathin 60 nm sections of the resin stub were cut using a Leica UC7 ultramicrotome. Sample preparation was performed at TEM-SEM Electron Microscopy Unit from Scientific and Technological Centers (CCiTUB), Universitat de Barcelona.

### Cell Viability

HeLa cells were seeded at 1 × 10^5^ cell/mL in a 96-well plate for 24 h before exposure. Serial dilutions of HAuCl_4_, NaAuS, and preformed Au NPs were added dropwise at a final concentration ranging from 25 to 1000 μM and gently homogenized. After 24 h, the culture medium was removed and replaced with a fresh medium to eliminate noninternalized NPs and minimize assay interference. Cell viability was then evaluated by using the resazurin-based PrestoBlue assay. Briefly, 10 μL of PrestoBlue reagent was added to each well, and plates were incubated for 2 h at 37 °C. Fluorescence was measured at λ_ex_ = 531 nm and λ_em_ = 572 nm using a Varioskan LUX microplate reader (Thermo Fisher Scientific). Absorbance measurements were not used due to potential overlap with the plasmonic absorption of Au NPs. All experiments were performed in triplicate, and data analysis was carried out using the OriginLab software.

### Synthesis of Au NPs

Following the method previously reported by Piella et al.,^47^ a 150 mL of freshly prepared reducing solution of sodium citrate (SC, 2.2 mM) containing 0.1 mL of tannic acid (TA, 2.5 mM) and 1 mL of potassium carbonate (K_2_CO_3_, 150 mM) was heated with a heating mantle in a 250 mL three-necked round-bottom flask under vigorous stirring. When the temperature reached 70 °C, 1 mL of tetrachloroauric acid (HAuCl_4_, 25 mM) was injected. The color of the solution changed rapidly to black-gray (less than 10 s) and then to orange-red in the following 1–2 min. The solution was kept at 70 °C for another 5 min to ensure complete reaction of the gold precursor. Immediately after the synthesis and in the same reaction vessel, the sample was diluted by extracting 55 mL and adding 55 mL of SC (2.2 mM). When the temperature reached again 70 °C, two injections of 0.5 mL of HAuCl_4_ (25 mM) on a time interval of 10 min were done.

### Effect of Intracellular ROS in the Cytoplasmic Reduction of Gold

Cells were seeded on a 10 mm Petri dish (for EM) or on an 8-well glass-bottom microslide (for CLSM) at 100.000 cell/cm^2^ and incubated overnight. For induced oxidative stress, 100 μM TBH was added to cell culture for 4 h, and then media were replaced. Then, HAuCl_4_ at a final concentration of 250 μM was added dropwise onto cell cultures and gently homogenized. After 24 h of incubation, samples were processed correspondingly and imaged by TEM and CLSM.

### Confocal Laser Scanning Microscopy (CSLM)

After 24 h of exposure, cells were labeled with CellMask and placed on the confocal microscope (37 °C, 5% CO_2_) for their observation. To image the Au NPs, the confocal laser scanning microscope (Spectral CLSM Olympus FV1000) was set to reflectance mode.^69^ For this, the dichroic mirror was retracted, only a T80/20 beam splitter was set instead, the reflection mode allowed, and the emission window was set at Δ15 nm relative to the laser source wavelength.

## Supplementary Material



## Data Availability

The data that support the findings of this study are available on request from the corresponding authors.

## Notes

Additional BF-TEM images of intracellular Au NP formation in HeLa cells exposed to HAuCl_4_ and NaAuS, characterization of citrate-stabilized Au NPs used in cytotoxicity assays, NP recovery after intracellular synthesis by cell lysis, EDX spectrum of NaAuS-treated cells, composition of cCCM, TEM images of extracellular Au NP formation, crystalline Au identification in extracellular products, and time-resolved UV–vis spectra of Au NP formation in cCCM and controls (PDF)
