# Supplementary material for: In Situ Mineralization of Gold Nanoparticles from Sodium Aurothiomalate or Tetrachloroauric Acid in Human Cells
Source: ACS Nanosci Au. 2026 Mar 10;6(3):469–81. doi: 10.1021/acsnanoscienceau.5c00199 (PMC13281188; doi:10.1021/acsnanoscienceau.5c00199)
Supplement: Supplementary file 1 [file ng5c00199_si_001.pdf]

Supplementary Information for

In Situ Mineralization of Gold Nanoparticles from Sodium Aurothiomalate or Tetrachloroauric Acid in Human Cells.

Muriel F. Gusta[1,2,3], Sofia Rubio[1], Macarena Cobaleda-Siles[2], Silvia Pujals[4], Maria de la Mata,[1,5] Jordi Arbiol[1,6], Neus G. Bastus[1,3*], Victor Puntes[1,2,3,6*]

[1] Catalan Institute of Nanoscience and Nanotechnology (ICN2), CSIC and BIST, Campus UAB, 08193 Bellaterra (Barcelona), Catalonia, Spain.
[2]Vall d'Hebron Institut de Recerca (VHIR), 08035, Barcelona, Spain
[3] Networking Research Centre for Bioengineering, Biomaterials and Nanomedicine (CIBER-BBN), 28029 Madrid, Spain
[4] Institute for Advanced Chemistry of Catalonia (IQAC-CSIC), Department of Biological Chemistry, 08034 Barcelona, Catalonia, Spain
[5]Dpto. Ciencia de los Materiales, I. M. y Q. I., IMEYMAT, Universidad de Cádiz, Campus Río San Pedro, 11510 Puerto Real, Spain
[6]ICREA, Pg. Lluís Companys 23, 08010 Barcelona, Catalonia, Spain

*Author for correspondence: neus.bastus@icn2.cat, victor.puntes@vhir.org

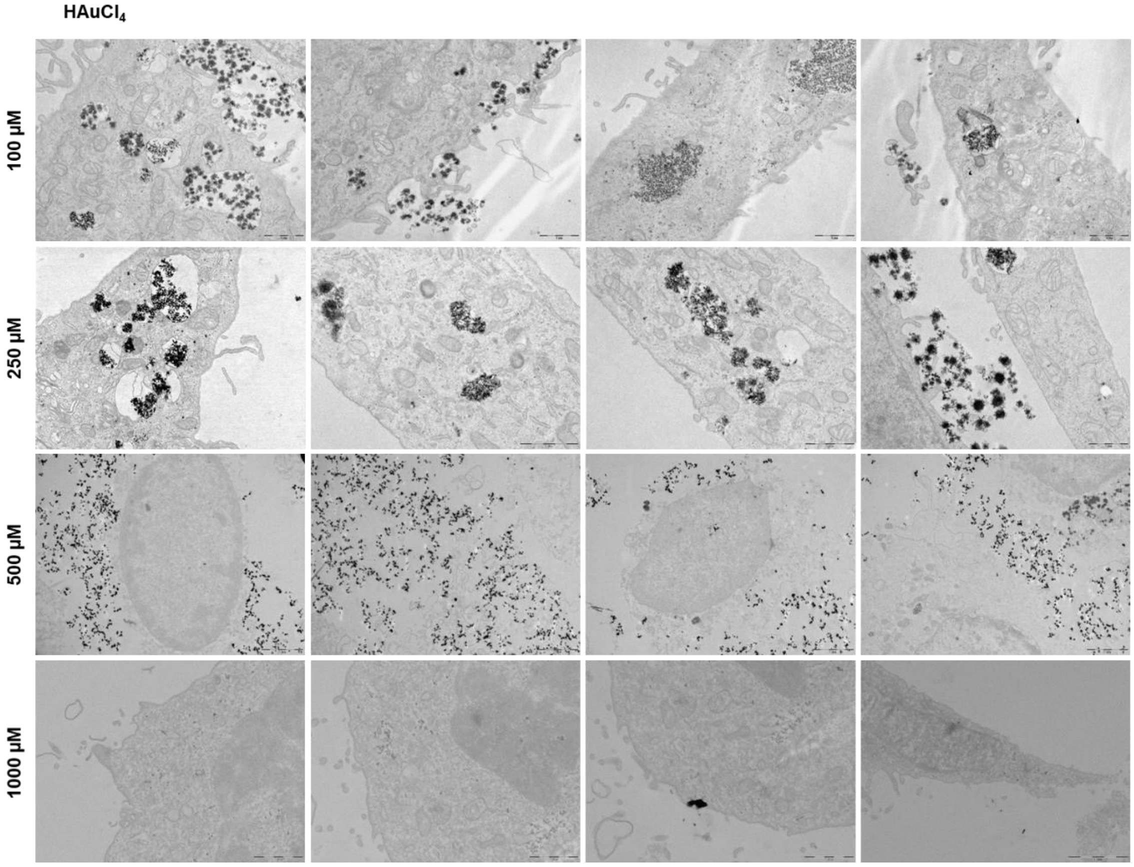

Figure S1. Representative Bright field Transmission Electron Microscopy (TEM) images of the intracellular formation of Au NPs resulting from the exposure of $HAuCl_4$ to HeLa cells at various precursor concentrations (100 µM, 250 µM, 500 µM, and 1000 µM) during a 24-hour incubation period.

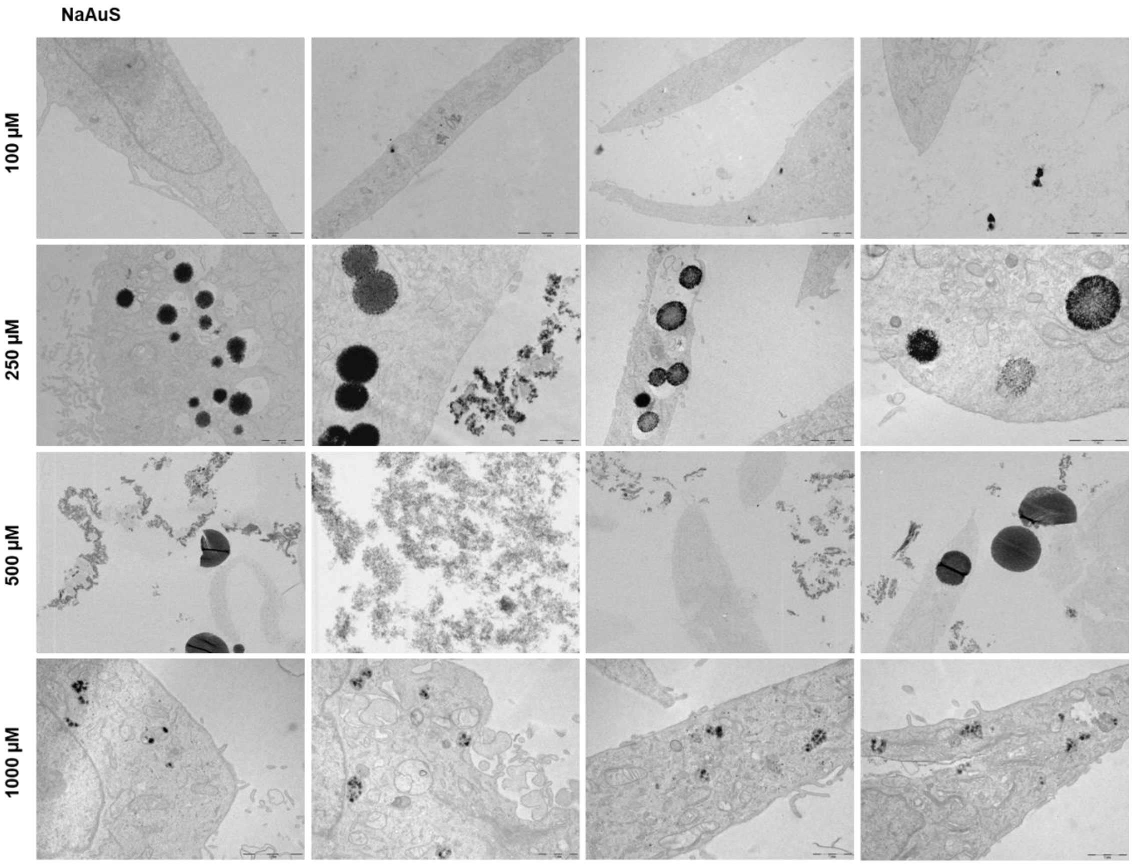

Figure S2. Representative Bright field Transmission Electron Microscopy (TEM) images of the intracellular formation of Au NPs resulting from the exposure of NaAuS to HeLa cells at various precursor concentrations (100 µM, 250 µM, 500 µM, and 1000 µM) during a 24-hour incubation period.

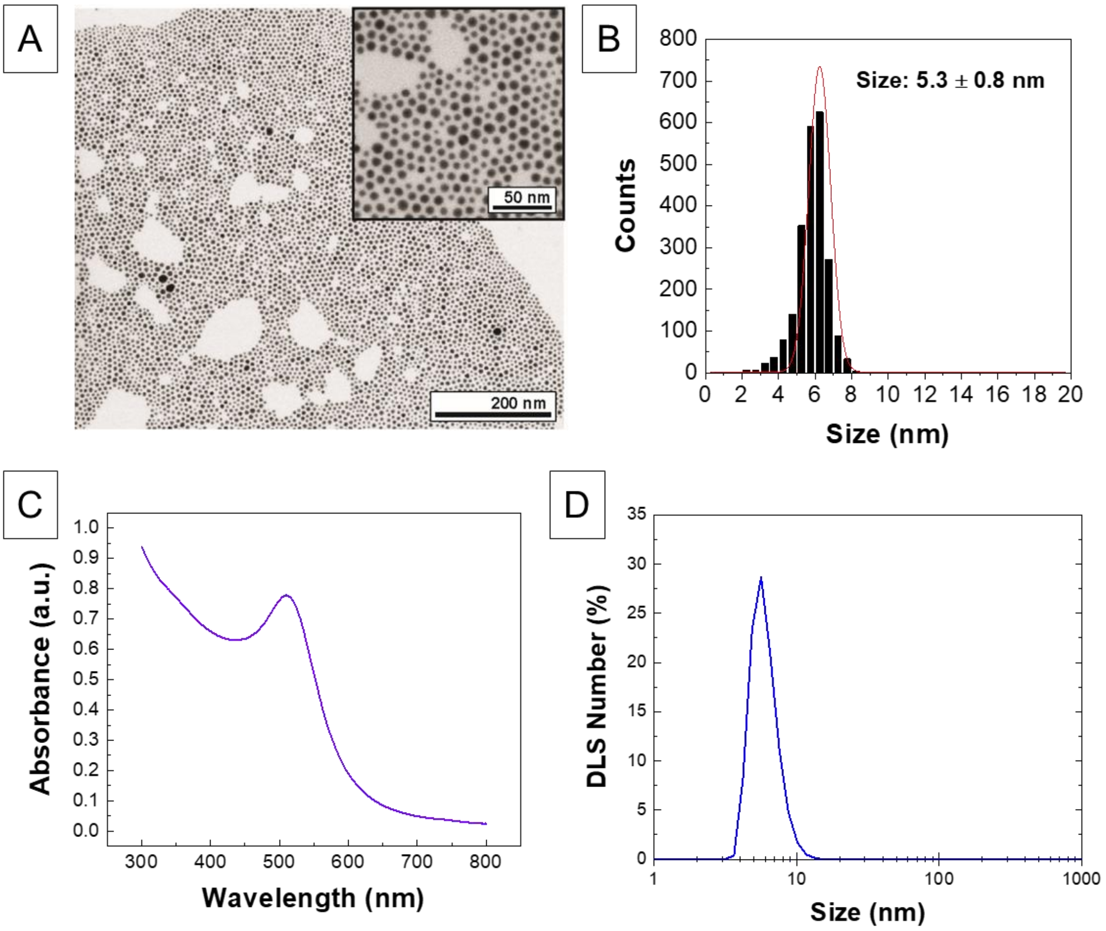

Figure S3. Characterization of citrate-stabilized Au NPs used in the cytotoxicity studies of Figure 1. (A) Representative Bright field TEM image of the synthesized Au NPs. **(B)** Size frequency distribution measured on the TEM images acquired. **(C)** UV-Vis spectra of the Au NPs as synthesized without any dilution, and **(D)** Dynamic Light Scattering size profile.

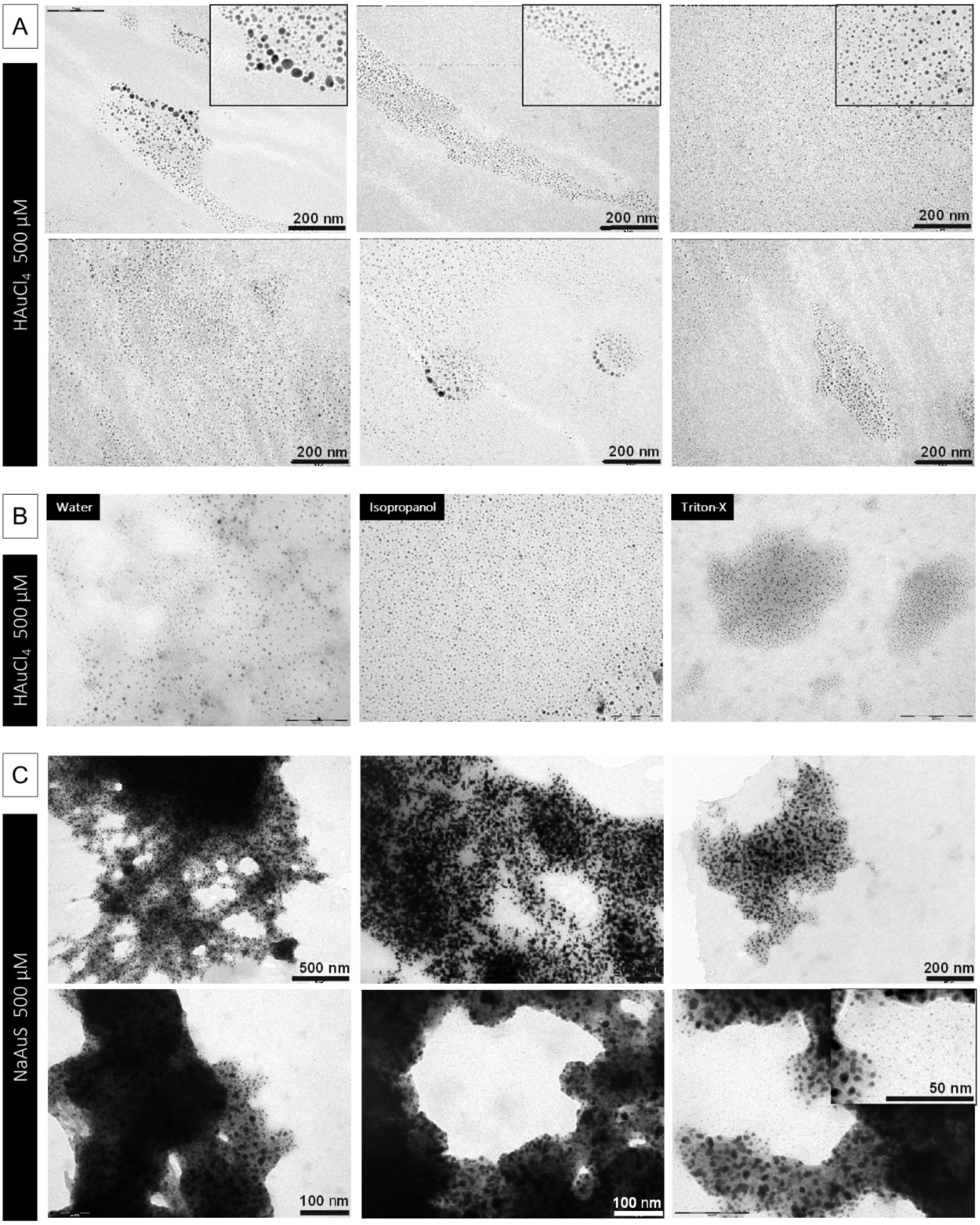

Figure S4. Intracellular Synthesis: NP recovery through cell lysis. At 24h of HeLa cells incubation with 500 µM $HAuCl_4$ or NaAuS, cells were centrifugated and separated from the supernatant and finally lysed with water. Resultant NPs from the lysate were characterized by Transmission Electron Microscopy (TEM). The formation of 2 nm Au NPs assessed by TEM, for different gold precursors at 500 µM **(A, C)** is observed. Control experiments show that NP morphology and yield extracted from cell lysate are independent of the cell lysis process **(B)**.

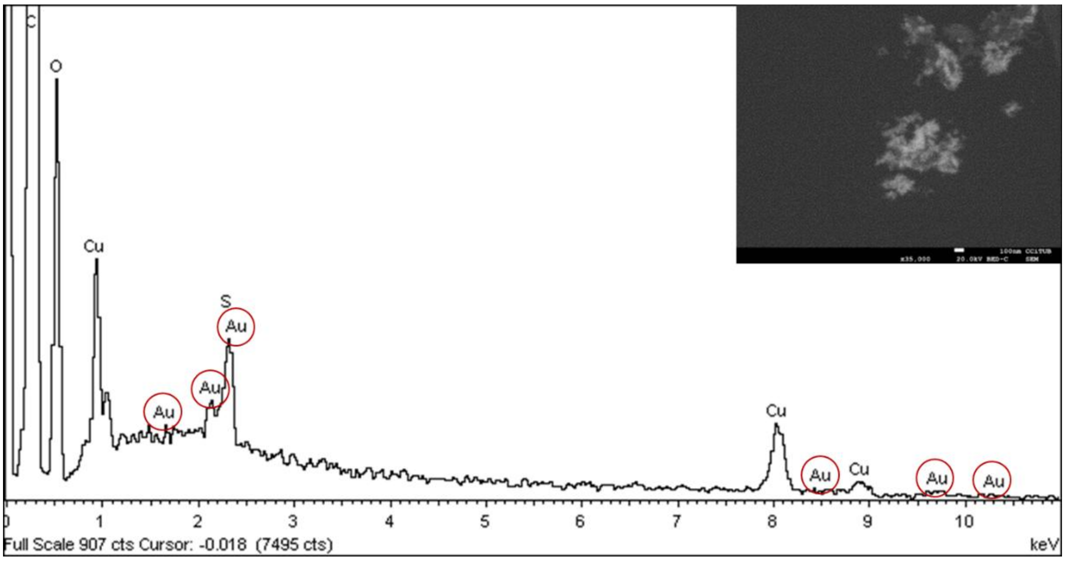

Figure S5. EDX spectrum of NaAuS precursor (250 µM) incubated in HeLa cell for 24h. An image of the sample region analyzed is shown on the upper right corner of the spectrum. Peaks detecting gold element are labelled in red color.

Table S1. Composition of completed cell culture media used to culture cell lines and expose Au precursors for extracellular synthesis. Refence 11965092 from ThermoFisher (Dulbecco, R. and Freeman, G. (1959) Virology 8:396).

Components	Molecular Weight	Concentration (mg/L)	mM
**Amino Acids**			
Glycine	75.0	30.0	0.4
L-Arginine hydrochloride	211.0	84.0	0.39810428
L-Cystine 2HCl	313.0	63.0	0.20127796
L-Glutamine	146.0	584.0	4.0
L-Histidine hydrochloride-H2O	210.0	42.0	0.2
L-Isoleucine	131.0	105.0	0.8015267
L-Leucine	131.0	105.0	0.8015267
L-Lysine hydrochloride	183.0	146.0	0.7978142
L-Methionine	149.0	30.0	0.20134228
L-Phenylalanine	165.0	66.0	0.4
L-Serine	105.0	42.0	0.4
L-Threonine	119.0	95.0	0.79831934
L-Tryptophan	204.0	16.0	0.078431375
L-Tyrosine disodium salt dihydrate	261.0	104.0	0.39846742
L-Valine	117.0	94.0	0.8034188
**Vitamins**			
Choline chloride	140.0	4.0	0.028571429
D-Calcium pantothenate	477.0	4.0	0.008385744
Folic Acid	441.0	4.0	0.009070295
Niacinamide	122.0	4.0	0.032786883
Pyridoxine hydrochloride	206.0	4.0	0.019417476
Riboflavin	376.0	0.4	0.0010638298
Thiamine hydrochloride	337.0	4.0	0.011869436
i-Inositol	180.0	7.2	0.04
**Inorganic Salts**			
Calcium Chloride (CaCl2) (anhyd.)	111.0	200.0	18.018.018
Ferric Nitrate (Fe(NO3)3"9H2O)	404.0	0.1	2,48E+03
Magnesium Sulfate (MgSO4) (anhyd.)	120.0	97.67	0.8139166
Potassium Chloride (KCl)	75.0	400.0	53.333.335
Sodium Bicarbonate (NaHCO3)	84.0	3700.0	4.404.762
Sodium Chloride (NaCl)	58.0	6400.0	110.344.826
Sodium Phosphate monobasic (NaH2PO4-H2O)	138.0	125.0	0.9057971
**Other Components**			
D-Glucose (Dextrose)	180.0	4500.0	25.0
Phenol Red	376.4	15.0	0.039851222

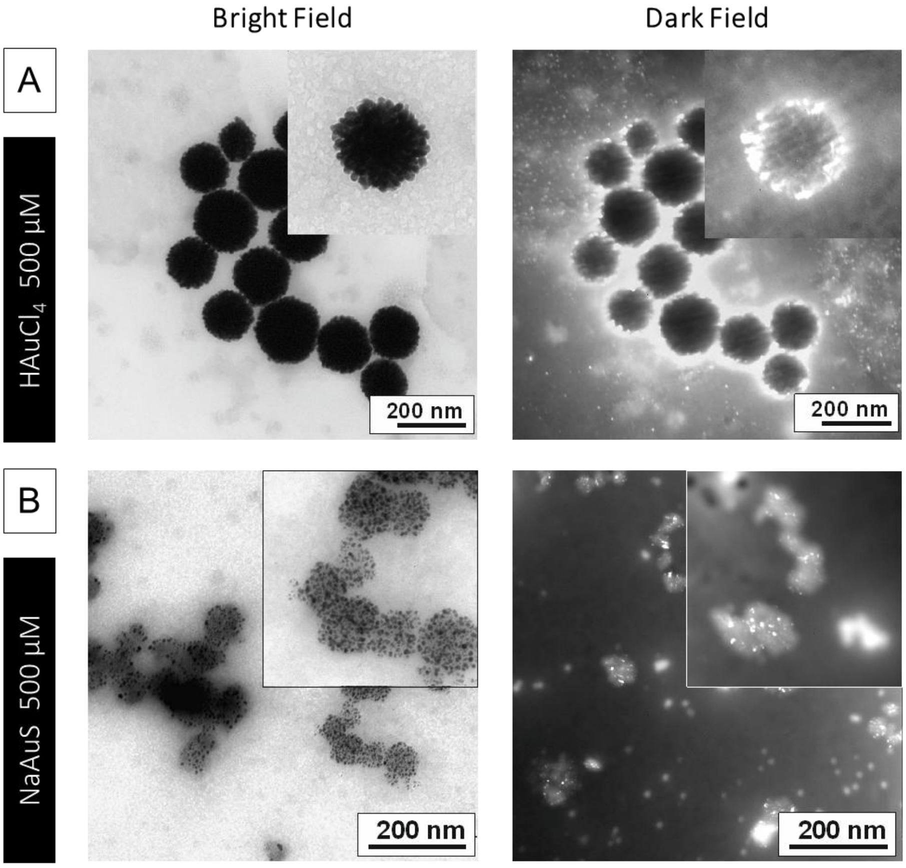

Figure S6. Representative Transmission Electron Microscopy (TEM) images of the extracellular formation of Au NPs resulting from the exposure of $HAuCl_4$ **(A)** and NaAuS **(B)** to cCCM at 500 µM during a 64-hour incubation period. Dark-field imaging and the high contrast of the Au NPs allowed us to easily distinguish the NP against organic matter, in particular proteins and other high-contrast species present in the CCM.

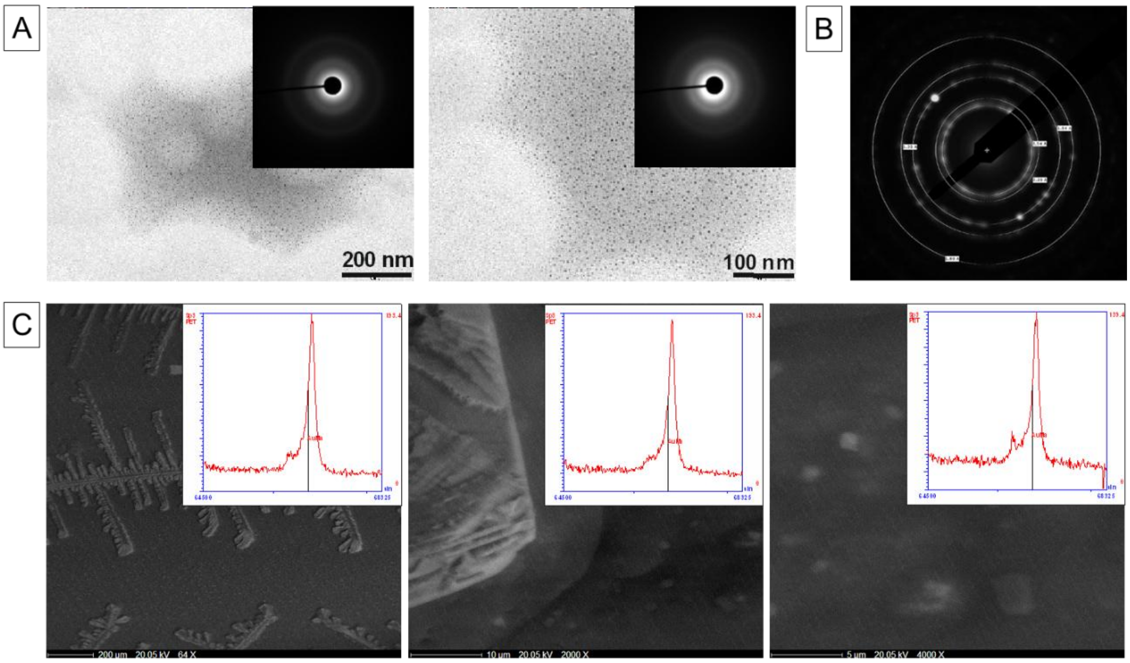

Figure S7. Identification of Au in the crystalline structures formed after 24h exposure of $HAuCl_4$ 500 µM to cCCM. Two independent selected areas of the sample show similar diffraction patterns. The selected area electron diffraction (SAED) pattern displays the crystalline structure of Au NPs **(A)**. Diffraction rings corresponds to different Au planes **(B)**. Electron Probe Microanalyzer (EPMA), a non-destructive analytical tool used to determine the chemical composition of solid materials, confirms the gold nature of the samples **(C).**

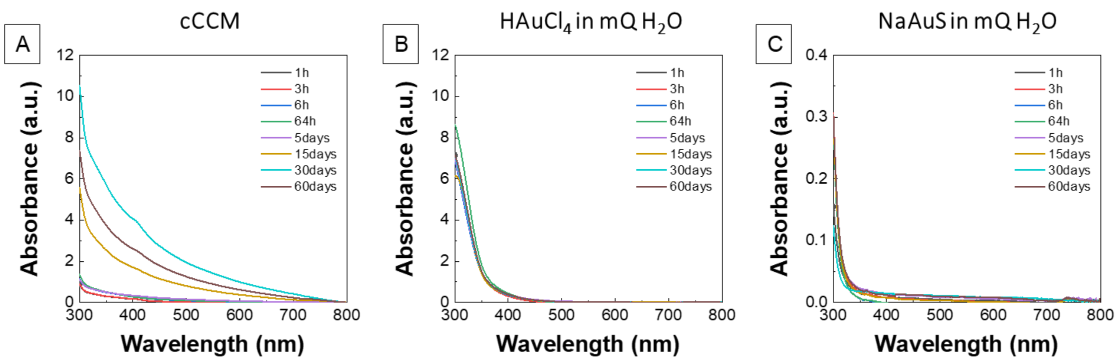

Figure S8. (A) Time-dependent UV−vis spectra of cCCM at 37°C for 60 days using Milli-Q water as the reference baseline. The progressive increase in UV-vis absorbance of the cCCM is attributed to progressive oxidation of the culture media with time. (**B**) UV–vis absorption spectra of $HAuCl_4$ and (**C**) NaAuS precursors in Milli-Q water monitored over 60 days.

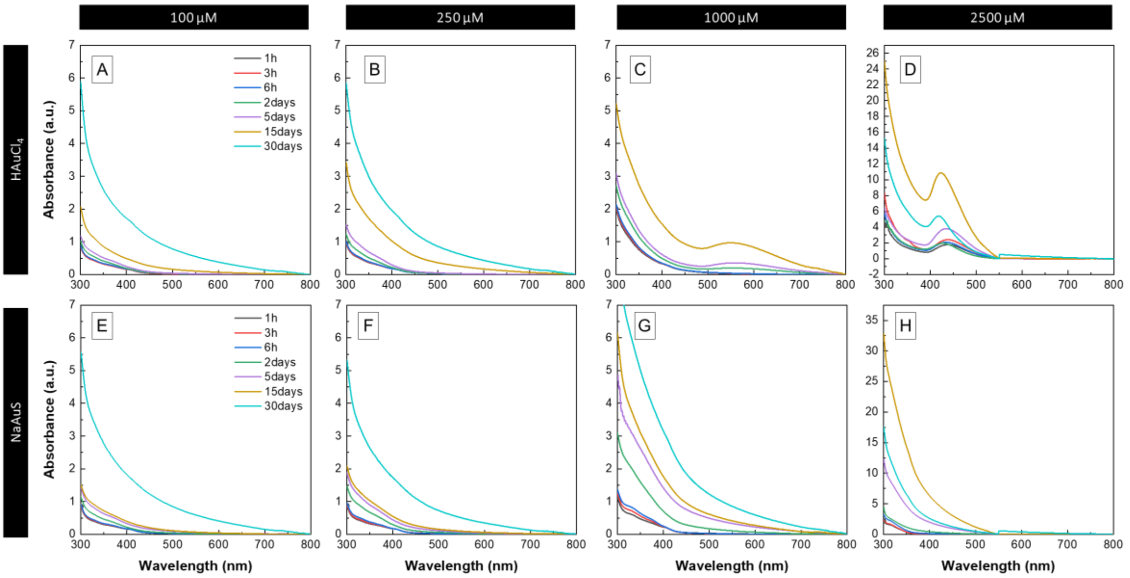

Figure S9. Temporal evolution of UV-Vis spectra of Au NPs formation after the incubation of $HAuCl_4$ **(A-D)** and NaAuS **(E-H)** precursors salt in in cCCM at a final concentration of 100-2500 µM.
